# The Influence of Family-Related Factors on Suicide in Major Depression Patients

**DOI:** 10.3389/fpsyt.2022.919610

**Published:** 2022-07-01

**Authors:** Wei Wang, Xin Guo, Lijun Kang, Nan Zhang, Simeng Ma, Jing Cheng, Li Fang, Zhongchun Liu

**Affiliations:** ^1^Department of Psychiatry, Renmin Hospital of Wuhan University, Wuhan, China; ^2^Department of Psychiatry, Jingmen No. 2 People’s Hospital, Jingmen, China

**Keywords:** depression, suicide, family, abuse, parenting style

## Abstract

**Objective:**

To explore the influence of family-related factors on suicide-related behaviors of patients with major depression disorder, and to provide scientific evidence for effective preventive measures.

**Methods:**

A total of 852 outpatients at Renmin Hospital of Wuhan University were enrolled in this cross-sectional study from April 2019 to January 2021. The general demographic information and family-related information of the patients were collected *via* a general information questionnaire, the Family Assessment Device, the Egna Minnen av Barndoms Uppfostran, and the Childhood Trauma Questionnaire.

**Results:**

Participants without suicide-related behaviors accounted for 10.45% of the total sample, those with suicidal ideation accounted for 47.54%, those with suicidal plans accounted for 24.77% and with suicidal attempts for 17.25%. Patients with major depression disorder who have lower education level, who were separated from their parents, who have severely impaired family function, who experienced childhood abuse, and whose parents adopted apathetic and severe child-rearing styles had a higher risk of suicide-related behaviors. In the multivariate regression model, degree of major depression disorder, education and child-rearing style were independent risk factors for suicide-related behaviors.

**Conclusion:**

Patients with major depression disorder who have been separated from their parents, have severely impaired family function, were abused in childhood or have been exposed to improper childrearing styles have a greater risk of suicide. Family-related factors play a predictive role in suicide in patients with major depression disorder. More attention should be paid to family-related factors to reduce the occurrence of suicidal ideation and attempt.

## Introduction

Suicide is the act of deliberately killing oneself, and has now become a serious public health problem. World Health Organization (WHO) reported more than 800,000 suicidal cases every year, still there are many more trying to commit suicide (1). In 10–19-year-olds adolescents, suicide has become the third leading cause of death worldwide (2). Adolescence is now a high-risk period for first-time suicidal attempt (3). But as suicide is often deemed as a multifactorial phenomenon, it is hard to evaluate and predict the suicide risk in adolescence.

Mental illness is the most significant trigger of suicide. Compared with healthy people, suicide-related behaviors (SRB) such as suicidal ideations (SI), suicidal plans (SP) and suicidal attempts (SA) (4) are more often seen in people with poor mental health (5), with affective disorder being a recognized risk factor for suicide (6). Major depression disorder (MDD) is a common mental illness, mainly manifested by upset, slowed thinking and decreased volitional activity (7). In clinical cases, suicide attempt often signals severe MDD. WHO statistics has it that approximately 322 million people in the world suffer from MDD, with approximately 54.81 million in China, accounting for about 4.2% of the total population (8). Almost 15% of people with MDD die by suicide and 80% of teenagers who committed suicide suffer from severe MDD (9, 10).

Previous studies have shown that there are many factors affecting SRB in patients with MDD, such as age, sex, severity of MDD, impairment of social function and family history of suicide (11). SRB is also related to psychotic symptoms, insomnia and other symptoms (12). Personality characteristics may also play an important role in the occurrence of MDD and suicide (13). Recently, it is found that alexithymia is associated with suicidal ideation and may be mediated by cholesterol level (14–16). Another research reported significant differences in the activity of the bilateral orbitofrontal cortex and dorsolateral prefrontal lobe between patients with suicide attempt and patients without suicide attempt, which are the main areas involved in cognitive and attempt patterns (17–19). Therefore, it is speculated that the occurrence of SRB is related to cognition and attempt patterns. Many previous studies have reported that negative experiences in childhood can affect physical and mental health (20). Among all the negative childhood experience variables, the degree of childhood abuse and family dysfunction are the two most commonly studied. A study of Chinese teenagers suggested that there is a significant correlation between physical abuse and SI (21). Donath et al. sought to explore the influence of parenting style and found that children who received little warmth and control from parents may be at high risk of suicide (22). However, due to the heterogeneity of research samples and designs, almost no conclusive evidence has been found. Therefore, more efforts should be paid to further examine the effects of family-related factors on SRB in patients with MDD. The Family Assessment Device (FAD), the Egna Minnen av Barndoms Uppfostran (EMBU), and the Childhood Trauma Questionnaire (CTQ) are often used to evaluate social and psychological factors in patients with MDD with respect to the living environment of patients, making it possible to conduct an in-depth study on the impact of the family environment on patients.

Although demographic data show that China’s suicide rate continues to decline, the rate of decline is slow, or even rebounding, so it is urgent to put forward targeted preventive measures (23). Suicide can have serious consequences, such as disability and death, which could bring great impact on individuals, families and society, finding out the influencing factors of SRB in patients with MDD can facilitate formulating measures to prevent suicide. In this way, the incidence of suicide can be effectively reduced and its adverse effects avoided.

We hypotheses that depressed patients with SRB were exposed to more and severe childhood negative life events. We predicted that a specific childhood negative life event may be an independent risk factor for suicidal ideation in depressed patients.

## Materials and Methods

### Participants

The experimental data were based on the “Early warning system and comprehensive intervention for depression” (ESCID) (24). All the respondents were outpatients being treated at Renmin Hospital of Wuhan University from April 2019 to January 2021. Experienced doctors diagnosed depressed patients according to diagnostic criteria of MDD in the 5th edition of the American Diagnostic and Statistical Manual of Mental Disorders (DSM-5). The inclusion criteria for this study were as follows: age 18–55 years old; junior high school education and above (to avoid that patients can’t understand the meaning of the questions in the questionnaires); the score of HAMD-17 >17 (to ensure that the patient is currently in depression episode and not in remission); signed informed consent. The exclusion criteria were as follows: Symptoms are too severe to cooperate in completing the questionnaire (such as stupor); pregnancy; history of organic brain disease, and alcohol or other addictive substance dependence (according to DSM-5). There were 852 patients who fulfilled the criteria for inclusion: 204 males and 648 females.

### Study Procedure

**FIGURE 1:**
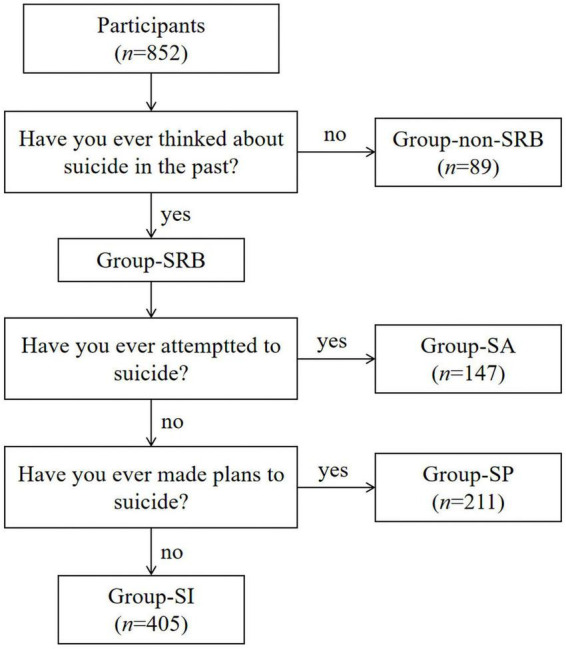
Flowchart representing procedure of dividing participants into groups.

Patients who met the inclusion and exclusion criteria were explained the purpose and process of the study and asked whether the patients were willing to join the group. Patients who gave informed consent were recruited in the study. After joining the study, patients needed to provide general information. Then we interviewed patients with Mini International Neuropsychiatric Interview (M.I.N.I.). According to the answer of question “Have you ever thinked about suicide in the past?, “they were divided into two groups, one group without SRB (group-non-SRB) while another group with SRB (group-SRB). According to the answer of another two questions “Have you ever made plans to suicide?” and “Have you ever attempted to suicide?,” group-SRB can be divided into three sub-groups which are group-SI, group-SP and group-SA (Figure 1). Then they would complete the FAD, EMBU and CTQ.

### Research Instruments

M.I.N.I. is a toolbox developed by Sheehan and Lecrubier to screen and diagnose the 16 kinds of axis-I psychiatric disorders and one kind of personality disorder simply, effectively and reliably (25). In this study, we used the Chinese version of M.I.N.I. which was proved to have high reliability and validity (26).

General information questionnaire: This questionnaire was self-designed by the researchers to assess demographic data, such as gender, age, place of residence, education level, marital emotional status and separation from parents.

FAD: This instrument includes seven subscales and a total of 60 items. The subscales are problem solving, communication, role, emotional response, emotional intervention, behavior control, and overall function. The questionnaire is used to identify problems in home systems simply and effectively. The lower the score for each item, the better the family relationship is (27). The Chinese version of FAD scale we used has good reliability and validity (28).

EMBU: This tool was developed by Swedish scholar Paris and others in 1980 to assess parental parenting styles and behaviors to determine the relationship between family environment and mental disorders (29). Here we used the version revised in China in 1993. In this version, the father’s parenting style consists of six subscales and the mother’s parenting style consists of five subscales. Father’s factor I (FI) means emotional warmth and understanding; Father’s factor II (FII) means abusive and punitive; Father’s factor III (FIII) means overinvolved; Father’s factor IV (FIV) means favoring patient; Father’s factor V (FV) means rejecting and denying; Father’s factor VI (FVI) means overprotective; Mother’s factor I (MI) means emotional warmth and understanding; Mother’s factor II(MII) means overinvolved and overprotective; Mother’s factor III (MIII) means rejecting and denying; Mother’s factor IV (MIV) means abusive and punitive; Mother’s factor V (MV) means favoring patient (30).

CTQ: Compiled by Bernstein et al., it is mainly used to assess the growth experience before the age of 16 with respect to emotional abuse, physical abuse, sexual abuse, emotional neglect and physical neglect. The higher the score is, the greater the abuse (31). The Chinese version we used in our study is a promising tool for assessing various subtypes of childhood adversities (32).

### Statistical Analysis

IBM SPSS Statistics (Version 26.0) was used to compare the demographic data and the scores of the scales. The count data were tested with the chi-squared test, and the data that did not meet the chi-squared test standard were analyzed *via* Fisher’s exact probability method. After normality test, we found that all the measurement data are non-normal distribution, so we use the Kruskal-Wallis test and percentile to show the results. Univariable binary logistic regression was used to explore the related factors between the group-non-SRB and the group-SRB with the data met the regressions assumptions. The independent influencing factors of the three kinds of SRB, which met the regressions assumptions, were further explored by multiple logistic regression analysis. The test level was α = 0.05 (two-tailed test), and the results were statistically significant when *P*<0.05.

## Results

### Socio-Demographic Characteristics

Participants without SRB accounted for 10.45% of the total sample, those with SI accounted for 47.54%, those with SP accounted for 24.77% and with SA for 17.25%. There were no significant differences in gender, place of residence or emotional status among the four groups (*P* > 0.05). There are general differences in education level among the four groups (χ^2^ = 18.794, *P* = 0.004). That whether patients were separated from their parents in childhood showed a significant difference among the four groups (χ^2^ = 8.018, *P* = 0.046). We also found significant differences in age distribution among the four groups (*H* = 11.829, *P* = 0.008). The scores of HAMD-17 were different among the four groups (*H* = 43.747, *P* < 0.001) (Supplementary Table 1).

**FIGURE 2:**
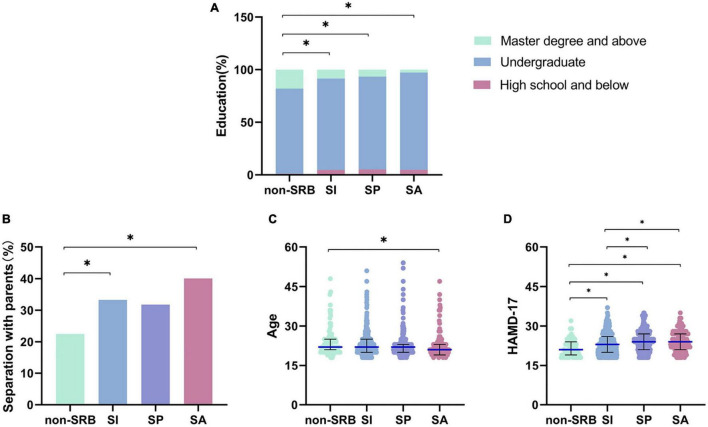
Pairwise comparison of Socio-demographic characteristics. **(A)** Education level in patients. **(B)** Separation with parents in patients. **(C)** Age in patients. **(D)** Severity of symptoms of depression in patients. *P< 0.05. Non-SRB, group without suicide-related behavior; SI, group with suicidal ideation; SP, group with suicidal plan; SA, group with suicidal attempt.

By making a pairwise comparison among the four groups in education, we found that there were significant differences between non-SRB and SI (χ^2^ = 8.395, *P* = 0.014), between non-SRB and SP (χ^2^ = 10.275, *P* = 0.005), and between non-SRB and SA (χ^2^ = 17.410, *P*<0.001). The higher the level of education, the higher the proportion of patients without SRB. However, the separation of parents only shows significant difference between non-SRB and SI (χ^2^ = 3.998, *P* = 0.046), and between non-SRB and SA (χ^2^ = 7.767, *P* = 0.005). The significant difference in age distribution only exists between non-SRB and SA (*H* = 106.495, *P* = 0.007). The results of pairwise comparison showed the scores of HAMD-17 were significant higher in patients with SI (*H* = –92.678, *P* = 0.007), SP (*H* = –182.075, *P*<0.001) and SA (*H* = –160.723, *P*<0.001) compared with those without SRB. When compared with the scores of HAMD-17 of patients with SI, the scores were higher in those with SP (*H* = –89.398, *P*<0.001) and SA (*H* = –68.046, *P* = 0.024) (Figure 2).

### Family-Related Factors

**FIGURE 3:**
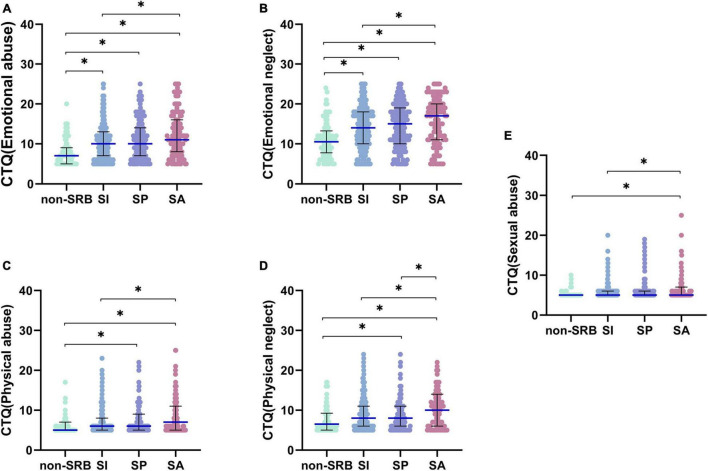
Pairwise comparison of childhood abuse. **(A)** Emotional abuse of childhood abuse. **(B)** Emotional neglect of childhood abuse. **(C)** Physical abuse of childhood abuse. **(D)** Physical neglect of childhood abuse. **(E)** Sexual abuse of childhood abuse. *P< 0.05. Non-SRB, group without suicide-related behavior; SI, group with suicidal ideation; SP, group with suicidal plan; SA, group with suicidal attempt.

According to the scores of each dimension in CTQ, we found that there were differences among the four groups in emotional abuse (*H* = 41.836, *P* < 0.001), physical abuse (*H* = 24.257, *P* < 0.001), sexual abuse (*H* = 15.828, *P* = 0.001), emotional neglect (*H* = 40.935, *P* < 0.001) and physical neglect (*H* = 19.669, *P* < 0.001) (Table 1). The results of pairwise comparison show that there are significant differences in emotional abuse between non-SRB and SI (*H* = –113.588, *P* < 0.001), between non-SRB and SP (*H* = –146.010, *P* < 0.001), between non-SRB and SA (*H* = –191.430, *P* < 0.001), between SI and SA (*H* = –77.843, *P* = 0.002); that in physical abuse between non-SRB and SP (*H* = –88.482,*P* = 0.009), between non-SRB and SA (*H* = –132.629, *P*<0.001), between SI and SA (*H* = –76.067, *P* = 0.002); that in sexual abuse between non-SRB and SA (*H* = –91.331, *P* = 0.001), between SI and SA (*H* = –54.175, *P* = 0.014); that in emotional neglect between non-SRB and SI (*H* = –118.786, *P* < 0.001), non-SRB and SP (*H* = –137.567, *P* < 0.001), non-SRB and SA (*H* = –194.082, *P* < 0.001), between SI and SA (*H* = –75.296, *P* = 0.004); and that in physical neglect between non-SRB and SI (*H* = –71.705, *P* = 0.043), between non-SRB and SA (*H* = –129.349, *P* < 0.001), between SI and SA (*H* = –57.644, *P* = 0.049), and between SP and SA (*H* = –76.203, *P* = 0.011) (Figure 3).

**TABLE 1:** Differences in CTQ, EMBU and FAD among the four groups.

	Non-SRB	SI	SP	SA	*H*	*P*
				
	Med (IQR)	Med (IQR)	Med (IQR)	Med (IQR)		
**CTQ (*n* = 744)**						
Emotional abuse	7 (5, 9)	10 (7, 13)	10 (7, 14)	11 (8, 16)	41.836	<0.001*
Physical abuse	5 (5, 7)	6 (5, 8)	6 (5, 9)	7 (5, 11)	24.257	<0.001*
Sexual abuse	5 (5, 5)	5 (5, 6)	5 (5, 6)	5 (5, 7)	15.828	<0.001*
Emotional neglect	10.5 (7.75, 13.25)	14 (10, 18)	15 (10, 19)	17 (11, 20)	40.935	<0.001*
Physical neglect	6.5 (5.00, 9.25)	8 (6, 11)	8 (6, 11)	10 (6, 14)	19.669	<0.001*
**EMBU (*n* = 324)**						
FI	44.5 (40.00, 52.25)	42 (34.5, 50.0)	40 (33.25, 45.75)	36 (30.5, 43.0)	16.550	0.001*
FII	17 (14.00, 20.25)	17 (14, 21)	17 (14.00, 23.75)	17 (14, 24)	1.225	0.747
FIII	20 (17.75, 24.25)	19 (17, 21)	20 (17, 22)	19 (16, 22)	4.866	0.182
FIV	12 (8.00, 15.25)	9 (6, 13)	8 (6.00, 11.75)	7 (5, 11)	13.076	0.004*
FV	8 (7, 12)	10 (8, 12)	10 (7.25, 12.00)	9 (8, 13)	1.854	0.603
FVI	10 (9.00, 12.25)	10 (8.5, 12.0)	10 (9, 13)	10 (8, 12)	0.715	0.870
MI	48 (40.75, 60.25)	47 (38, 53)	46 (37, 53)	38 (31.5, 47.0)	17.097	0.001*
MII	35 (30.75, 40.00)	34 (31.0, 39.5)	36 (30.25, 40.00)	36 (30.5, 41.0)	0.680	0.878
MIII	13 (10, 17)	14 (10, 16)	14 (11, 18)	14 (12, 18)	5.965	0.113
MIV	11.5 (10, 15)	13 (10, 17)	12 (10, 16)	15 (11.5, 20.5)	7.988	0.046*
MV	11.5 (8, 15)	10 (8.0, 12.5)	9 (7, 12)	8 (7, 11)	13.942	0.003*
**FAD (*n* = 418)**						
Problem solving	13 (12, 15)	14 (12, 16)	14.5 (13, 16)	15 (13, 16)	8.675	0.034*
Communication	21 (18, 25)	24 (21.0, 26.5)	24 (22, 27)	24 (22.00, 27.75)	11.910	0.008*
Role	24 (22, 27)	26 (24, 29)	26 (24.25, 29.00)	27 (25, 29)	15.722	0.001*
Emotional response	16 (14, 18)	17 (15, 18)	17 (15.00, 18.75)	17 (15, 18)	2.271	0.518
Emotional interaction	17 (15.5, 18.5)	17 (16, 18)	17 (16, 19)	18 (17, 20)	13.979	0.003*
Behavior control	21 (20, 23)	22 (21, 23)	22 (21, 23)	22 (20, 24)	2.104	0.551

*P < 0.05. Non-SRB, group without suicide-related behavior; SI, group with suicidal ideation; SP, group with suicidal plan; SA, group with suicidal attempt.

*F I, factor I of father; F II, factor II of father; F III, factor III of father; F IV, factor IV of father; F V factor V of father; F VI, factor VI of father; M I, factor I of mother; M II, factor II of mother; M III, factor III of mother; M IV, factor IV of mother; M V, factor V of mother.*

**FIGURE 4:**
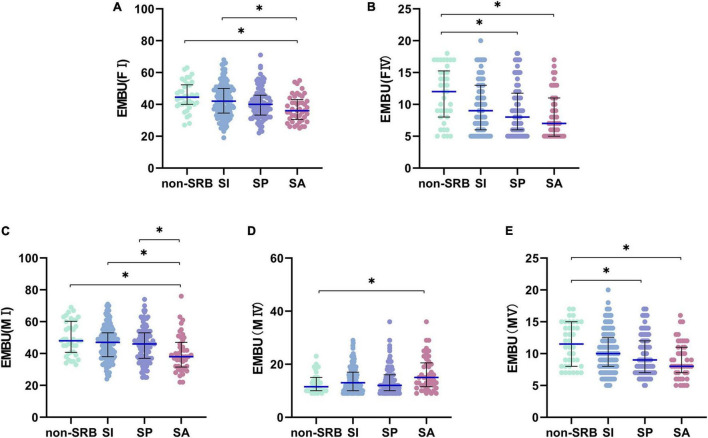
Pairwise comparison of EMBU. **(A)** Factor I of father. **(B)** Factor IV of father. **(C)** Factor I of mother. **(D)** Factor IV of mother. **(E)** Factor V of mother. *P< 0.05. Non-SRB, group without suicide-related behavior; SI, group with suicidal ideation; SP, group with suicidal plan; SA, group with suicidal attempt.

Regarding the EMBU, Only FI (*H* = 16.550,*P* = 0.001), FIV (*H* = 13.076,*P* = 0.004), MI (*H* = 17.097,*P* = 0.001), MIV (*H* = 7.988,*P* = 0.046) and MV (*H* = 13.942,*P* = 0.003) had significant differences among the four groups (Table 1). Further comparison shows that FI is different only between non-SRB and SA (*H* = 77.190,*P* = 0.001), SI and SA (*H* = 49.384,*P* = 0.012), FIV between non-SRB and SP (*H* = 54.061,*P* = 0.014), non-SRB and SA (*H* = 69.599,*P* = 0.004), MI between non-SRB and SA (*H* = 79.707,*P* = 0.001), SI and SA (*H* = 55.449,*P* = 0.003), SP and SA (*H* = 46.653,*P* = 0.033), MIV only between non-SRB and SA (*H* = –54.835,*P* = 0.046), MI between non-SRB and SP (*H* = 49.447,*P* = 0.032), and between non-SRB and SA (*H* = 70.637,*P* = 0.004) (Figure 4).

**FIGURE 5:**
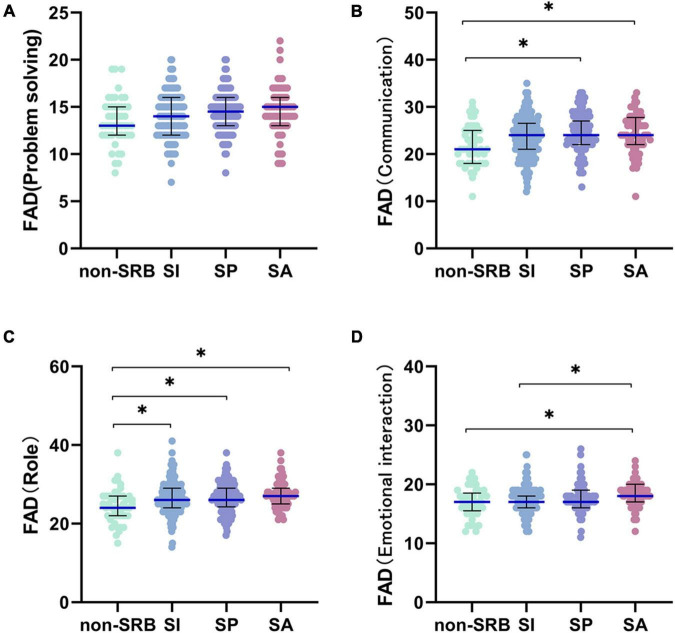
Pairwise comparison of FAD. **(A)** Problem solving of family function. **(B)** Communication of family function. **(C)** Role of family function. **(D)** Emotional interaction of family function. *P< 0.05. Non-SRB, group without suicide-related behavior; SI, group with suicidal ideation; SP, group with suicidal plan; SA, group with suicidal attempt.

There were significant differences in problem solving (*H* = 8.675,*P* = 0.034), communication (*H* = 11.910,*P* = 0.008), role (*H* = 15.722,*P* = 0.001), emotional intervention (*H* = 13.979,*P* = 0.003) based on the FAD scores (Table 1). Through pairwise comparison among the four groups, we found that there was no significant difference in problem solving among the four groups after Bonferroni correction, but there were significant differences in communication between non-SRB and SP (*H* = –65.217,*P* = 0.023), between non-SRB and SA (*H* = –67.307,*P* = 0.006), and roles between non-SRB and SI (*H* = –61.017,*P* = 0.010), between non-SRB and SP (*H* = –64.778,*P* = 0.009), between non-SRB and SA (*H* = –86.765,*P* = 0.001), and emotional intervention only between non-SRB and SA (*H* = –65.061,*P* = 0.022), and between SI and SA (*H* = –58.472,*P* = 0.003) (Figure 5).

### Binary Logistic Regression Analysis

In order to find out the independent influencing factors of SRB in patients with MDD, we conducted binary logistic regression between group-non-SRB and group-SRB. We have advanced single factor analysis to filter out the required variables. The results showed that the score of HAMD-17 (*OR* = 1.158, *P* = 0.013), education level (*OR* = 3.525, *P* = 0.010) and FIV in EMBU (*OR* = 0.796, *P* = 0.022) were independent risk factors for SRB. With the education level of master or above as a reference, patients who are undergraduates are 3.525 times more likely to develop SRB (*OR* = 3.525, *P* = 0.003) (Table 2).

**TABLE 2:** Binary logistic regression analysis between non-SRB and SRB.

	*OR* (95%*CI*)	*P*
HAMD-17	1.158(1.031∼1.301)	0.013
Education		0.032
High school and below	4.930(0.472∼51.511)	0.183
Undergraduate	3.525(1.347∼9.227)	0.010
**CTQ**		
Emotional abuse	1.024(0.887∼1.182)	0.751
Physical abuse	1.094(0.907∼1.320)	0.350
Emotional neglect	0.983(0.880∼1.098)	0.761
**EMBU**		
FI	1.016(0.949∼1.089)	0.646
FIV	0.805(0.668∼0.968)	0.021
MI	0.980(0.910∼1.055)	0.593
MV	1.150(0.893∼1.480)	0.279
**FAD**		
Problem solving	0.958(0.758∼1.212)	0.723
Communication	1.045(0.918∼1.189)	0.510
Role	1.115(0.984∼1.262)	0.087

*Non-SRB, group without suicide-related behavior; SRB, group with suicide-related behavior, including suicidal ideation, suicidal plan and suicidal attempt.*

### Multiple Logistic Regression Analysis

To explore the influence factors affecting the kind of SRB, we did multiple logistic regression analysis in three groups of SI, SP and SA after single factor difference analysis. The results showed that only the score of HAMD-17 (*OR* = 0.917, *P* = 0.042) is independent risk factors for the kind of SRB (Table 3).

**TABLE 3:** Multiple logistic regression analysis among SI, SP and SA.

(Ref*: SI)		*OR* (95%*CI*)	*P*
SP	HAMD-17	1.102(1.029∼1.180)	0.005
	CTQ		
	Physical abuse	1.038(0.948∼1.137)	0.421
	Sexual abuse	0.972(0.864∼1.093)	0.637
	Emotional neglect	0.982(0.912∼1.056)	0.619
	EMBU		
	FM	0.973(0.931∼1.018)	0.234
	M.	1.025(0.984∼1.069)	0.233
	M.	0.934(0.844∼1.034)	0.189
	FAD		
	Emotional interaction	1.080(0.949∼1.229)	0.243
SA	HAMD-17	1.024(0.934∼1.123)	0.616
	CTQ		
	Physical abuse	1.055(0.948∼1.174)	0.328
	Sexual abuse	1.012(0.893∼1.146)	0.853
	Emotional neglect	1.023(0.930∼1.126)	0.639
	EMBU		
	FM	0.981(0.926∼1.041)	0.533
	M.	0.980(0.927∼1.036)	0.477
	M.	0.962(0.827∼1.119)	0.611
	FAD		
	Emotional interaction	1.152(0.977∼1.358)	0.093

*Reference group: SI. SI, suicidal ideation; SP, suicidal plan; SA, suicidal attempt.

## Discussion

A total of 852 patients with MDD were enrolled in this study. Based on their questionnaire scores, the patients were divided into two groups: group-non-SRB (*n* = 89) and group-SRB (*n* = 763). The group-SRB was divided into three subgroup, namely, group-SI (*n* = 405), group-SP (*n* = 211), and group-SA (*n* = 147). The rate of SRB occurrence among the patients with MDD was 89.55%, of which SA account for 17.25%, which was slightly higher than what previous studies reported. The rate of SI was 47.54%, which was much higher than previous studies. Group-SP account for 24.77% (33, 34). Such a composition may be related to the sample size and whether the patients tell the truth, since some of MDD patients may conceal the truth because of a sense of shame. In terms of the sample size, however, the high SRB rate suggested that we should pay more attention to SRB in patients with MDD and take effective measures to prevent serious consequences, such as dead by suicide.

A recent ecological study in Mexico compared areas with high and low suicide rates and found no significant difference in education level between the two groups (35). Another large-scale study in southern Iran shows that people who commit suicide by self-immolation have lower education level (36). A cross-sectional study in Indonesia shows that people with lower education are more likely to suffer from MDD, while people with MDD have a higher suicide rate than healthy people (37). Our results are roughly the same as the above conclusions, the lower the level of education, the more likely patients with MDD to develop SRB, or even become an independent risk factor for SRB, but there is no significant impact on the type of SRB. As we all know, education plays a very important role in personal and social development. Education is closely related to the development of cognitive function, and also affects the economy, politics and culture of society, which can affect the incidence of SRB (38, 39).

Previous studies have reported that the hospitalization rate of children with mental illness is closely related to the family structure; specifically, the disruption of the family structure that affects children’s mental health (40). Our analysis on the separation from parents is also supportive to this conclusion by finding that patients with a history of separation with parents are more likely to develop SI and SA. On the contrary, teenagers who live with their biological parents are less likely to commit suicide than those who live with single parents, in foster care or alone, according to studies conducted in South Korea, Norway and elsewhere (41, 42).

Joiner’s suicide theory holds that habitual pain and fear tend to cause suicide risk, and childhood abuse is an noticeable risk factor for suicide (43). Research by Talmon et al. shows that emotional abuse may adversely affect the mental health of adults when their identity and self are challenged at certain times in the life cycle (44). Our study also found that all the emotional abuse, physical abuse, sexual abuse, emotional neglect and physical neglect revealed by the CTQ curvy were significantly different among the four groups and positively correlated with the occurrence of SRB. The mechanism of which may be related to the development of negative cognitive style caused by childhood abuse (45). Children who have experienced traumatic events are more likely to have problems in emotional and behavioral self-regulation in later life (46). In particular, the biological processes of children who have experienced traumatic events during stress will destroy the early development of the central nervous system, which may have an adverse impact on future brain function, so they are more likely to injure themselves and attempt suicide (47). However, people who reported SRB may also have a more negative view of themselves and their past than those who did not report SRB, increasing the likelihood of the former reporting negative childhood experiences.

In terms of the EMBU, our study showed that there were significant differences between FI and FIV and MI, MIV and MV among the four groups. Specifically, the scores of FI, FIV, MI and MV were negatively correlated to SRB while the score of MIV positively, which indicated that the more emotional warmth, understanding and favor for patient from parents and less abusive and punitive from mother, the lower the risk of SRB. Our result is consistent with previous studies on the finding that parents’ understanding of adolescents’ problems and concerns is a protective factor against SRB (48). Alas, some studies have shown that a lack of parental warmth and care and parental control of children with negative psychology such as criticism and guilt induction are related to patients’ depression and negative cognitive style (49). And parental overprotection in childhood may as well increase the risk of cognitive impairment, and neurocognitive impairment may be a risk factor for SRB (50, 51). Therefore, to mitigate the risk of SRB, adjusting the appropriate rearing style and improving the communication between parents and children were suggested.

According to the Health Behavior in School-aged Children (HBSC) survey, a multinational study performed by the World Health Organization, families play an important role in the lives of teenagers (52). Good parent-child relationships and communication are the key determinants of good mental health and positive behavior in adolescents (53). Previous studies have shown that broken families were more pront to SA occurrence or other risky behaviors among teenagers (54). The results of this study show that the FAD concepts problem solving, communication, role and emotional intervention have a certain influence on the occurrence of SRB. This means when the division of labor and completion of family members worsen off, and domestic care and attention decrease, patients are more likely to develop SA. Such finding is consistent with the previous studies, which suggested that adolescents who communicate openly with their parents have better adaptability, that parental support and emotional support can effectively prevent SA, and that active family processes play a vital role in protecting adolescents (55). Family dysfunction is not only related to adolescent suicide but is also considered to be a risk factor for suicide in old age, because when family functioning fails, the quality of care and quality of life of the elderly cannot be guaranteed (56–58).

Previous studies have shown that being female, being unmarried, living alone, being young, and having a low education level are factors that increase the risk of SRB (59–61). However, our data analysis showed no statistically significant differences in gender, residence and marital emotional status among group-non-SRB, group-SI, group-SP and group-SA, which may be due to the conditions under which the sample was recruited. Most previous studies were conducted in the general population. The purpose of this study was to screen out patients with confirmed MDD from psychiatric outpatients. There were significant differences in age distribution among the four groups. Specifically, the ages of those in SA were significantly lower than other groups. However, the results of previous studies are inconsistent. Some showed a lower age in the suicide group than the non-suicide group, while others showed that the incidence of SRB increased with age, which may be due to the different inclusion criteria used for the samples (62, 63). This difference may also be related to the different grouping methods used in each study. In terms of residence, the results of the study show that there is no significant difference among the four groups. However, most previous studies suggest that patients living in rural areas are more likely to commit suicide (64). This difference may be related to that the population of patients who visit the hospital are less from rural which lead to the bias of sample collection; some rural patients may not be able to see a doctor in time because of economic problems and lack of disease awareness.

The patients with MDD included in this study were not specifically classified, but many previous studies suggested that different subtypes of MDD could also affect SRB. Previous studies have suggested that MDD patients with melancholic features have a higher risk of suicide than patients without melancholic features (65). Another study similarly proved that the risk of suicide is higher in patients with psychotic depression (66). A cohort study suggests that among MDD patients treated with electroconvulsive therapy, patients with senile depression and psychotic depression have a significantly decreased risk of suicide (67). In addition, dissociative depression reports more SI and SA (68). An immunological study found that depression patients with high suicide risk showed significant immune abnormalities, suggesting a possible subtype of MDD (69). Therefore, in further research in the future, we will pay more attention to the relationship between subtypes of MDD and SRB.

## Limitations

There are still limitations to this study. Firstly, sample selection was not comprehensive enough. Most of the participants were from cities, participants were only 18–55 years old. The education level was limited to a certain extent, which may result in a certain degree of bias in the results. Secondly, patient compliance was not carefully monitored. Some patients selectively completed the scale, which could reduce the true sample size for some of the data. Thirdly, as most of the scales were self-rating scales, the authenticity of the data cannot be verified, and there may be discrepancies between the analysis results and the real situation. However, the total sample size was large, and the study was implemented with a standardized process. The results of the study still have a certain clinical significance.

## Conclusion

In this study, the probability of SRB in MDD patients was as high as 89.55%. Our results show that patients with MDD who have lower education level, who were separated from their parents, who have severely impaired family function, who experienced childhood abuse, and whose parents adopted apathetic and severe childrearing styles have a higher risk of SRB. Among them, the severity of depressive symptoms, education level and father’s parenting style even became independent risk factors of SRB. Therefore, to prevent SRB, greater attention should be paid to reducing family and school violence in order to reduce childhood abuse and improve the family structure and atmosphere. We believe that according to the conclusion of the study, the rate of SRB will be greatly reduced by proposing and implementing effective solutions.

## Data Availability Statement

The raw data supporting the conclusions of this article will be made available by the authors, without undue reservation.

## Ethics Statement

The studies involving human participants were reviewed and approved by Renmin Hospital of Wuhan University. The patients/participants provided their written informed consent to participate in this study.

## Author Contributions

All authors listed have made a substantial, direct, and intellectual contribution to the work, and approved it for publication.

## Conflict of Interest

The authors declare that the research was conducted in the absence of any commercial or financial relationships that could be construed as a potential conflict of interest.

## Publisher’s Note

All claims expressed in this article are solely those of the authors and do not necessarily represent those of their affiliated organizations, or those of the publisher, the editors and the reviewers. Any product that may be evaluated in this article, or claim that may be made by its manufacturer, is not guaranteed or endorsed by the publisher.
