# Supplementary material for: The Influence of Family-Related Factors on Suicide in Major Depression Patients
Source: Front Psychiatry. 2022 Jul 1;13:919610. doi: 10.3389/fpsyt.2022.919610 (PMC9283679; doi:10.3389/fpsyt.2022.919610)
Supplement: Supplementary file 1 [file Table_1.DOCX]

Table 1 Differences in Socio-demographic characteristics among the Four Groups

|  | non-SRB | SI | SP | SA | *χ2/H* | *P* |
| --- | --- | --- | --- | --- | --- | --- |
|  | (*n*=89) | (*n*=405) | (*n*=211) | (*n*=147) |  |  |
| Gender (N,%) | | | | | | |
| female | 68(10.5) | 307(47.4) | 158(24.4) | 115(47.7) | 0.562 | 0.905 |
| male | 21(10.3) | 98(48.0) | 53(26.0) | 32(15.7) |  |  |
| Residence (N,%) | | | | | | |
| countryside | 3(6.8) | 22(50.0) | 11(25.0) | 8(18.2) | 3.542 | 0.742^a^ |
| town | 13(10.4) | 66(52.8) | 24(19.2) | 22(17.6) |  |  |
| city | 73(10.8) | 314(46.4) | 176(26.0) | 114(16.8) |  |  |
| Marital emotional status (N,%) | | | | | | |
| single | 60(10.3) | 269(46.3) | 151(26.0) | 101(17.4) | 4.015 | 0.675 |
| in love | 19(9.2) | 104(50.5) | 48(23.3) | 35(17.0) |  |  |
| married | 10(15.4) | 32(49.2) | 12(18.5) | 11(16.9) |  |  |
| Education (N,%) | | | | | | |
| High school and below | 1(2.6) | 19(50) | 11(28.9) | 7(18.4) | 18.794 | 0.004^a*^ |
| Undergraduate | 72(9.7) | 352(47.2) | 186(24.9) | 136(18.2) |  |  |
| Master degree and above | 16(23.5) | 34(50) | 14(20.6) | 4(5.9) |  |  |
| Separation with parents (N,%) | | | | | | |
| no | 69(12.1) | 270(47.3) | 144(25.2) | 88(15.4) | 8.018 | 0.046^*^ |
| yes | 20(10.4) | 135(47.5) | 67(24.8) | 59(17.3) |  |  |
| Age  (Med, IQR) | 22（21,25） | 22（20,25） | 22（20,23） | 21(19,23) | 11.829 | 0.008^*^ |
| HAMD-17  (Med, IQR) | 21（19,24） | 23（20,26） | 24（21,27） | 24(21,27) | 43.747 | ＜0.001^*^ |

a: Fisher's exact test; *: *P*<0.05; Non-SRB: group without suicide-related behavior; SI: group with suicidal ideation; SP: group with suicidal plan; SA: group with suicidal attempt
